# S100-EPISPOT: A New Tool to Detect Viable Circulating Melanoma Cells

**DOI:** 10.3390/cells8070755

**Published:** 2019-07-20

**Authors:** Laure Cayrefourcq, Aurélie De Roeck, Caroline Garcia, Pierre-Emmanuel Stoebner, Fanny Fichel, Françoise Garima, Françoise Perriard, Jean-Pierre Daures, Laurent Meunier, Catherine Alix-Panabières

**Affiliations:** 1 Laboratory of Rare Human Circulating Cells (LCCRH), University Medical Centre of Montpellier, UPRES EA2415, 34093 Montpellier, France; 2 Department of Dermatology, Nîmes University Hospital, University of Montpellier, 30029 Nîmes, France; 3 UPRES EA2415, University Institute of Clinical Research (IURC), Montpellier University, 34093 Montpellier, France

**Keywords:** circulating tumor cells, melanoma, liquid biopsy, EPISPOT, CellSearch^®^

## Abstract

Metastatic melanoma is one of the most aggressive and drug-resistant cancers with very poor overall survival. Circulating melanoma cells (CMCs) were first described in 1991. However, there is no general consensus on the clinical utility of CMC detection, largely due to conflicting results linked to the use of heterogeneous patient populations and different detection methods. Here, we developed a new EPithelial ImmunoSPOT (EPISPOT) assay to detect viable CMCs based on their secretion of the S100 protein (S100-EPISPOT). Then, we compared the results obtained with the S100-EPISPOT assay and the CellSearch^®^ CMC kit using blood samples from a homogeneous population of patients with metastatic melanoma. We found that S100-EPISPOT sensitivity was significantly higher than that of CellSearch^®^. Specifically, the percentage of patients with ≥2 CMCs was significantly higher using S100-EPISPOT than CellSearch^®^ (48% and 21%, respectively; *p* = 0.0114). Concerning CMC prognostic value, only the CellSearch^®^ results showed a significant association with overall survival (*p* = 0.006). However, due to the higher sensitivity of the new S100-EPISPOT assay, it would be interesting to determine whether this functional test could be used in patients with non-metastatic melanoma for the early detection of tumor relapse and for monitoring the treatment response.

## 1. Introduction

Melanoma is the most malignant skin cancer, and its incidence rate is increasing worldwide. Early stage and localized melanoma can be cured by surgical resection. Conversely, metastatic melanoma is one of the most aggressive and drug-resistant cancers with very poor overall survival (OS) (six to nine months). Melanoma management has recently undergone revolutionary changes with the discovery of predictive tumor biomarkers (BRAF mutations and immune checkpoint inhibitors such as programmed cell death protein 1 (PD-1), its ligand (PD-L1), and cytotoxic T-lymphocyte antigen 4 (CTLA-4)) and the development of the associated treatments. These new treatments, alone or in combination, have dramatically improved the outcome of patients with metastatic melanoma. For example, the anti-PD-1 drug pembrolizumab has demonstrated benefits to progression-free survival (34–38%) and objective responses (21–25%) at six months compared with chemotherapy (16% and 4%; *p* < 0.0001) [1]. However, despite the good response rates, immunotherapy results in systemic toxicity, and it is not effective in all patients.

Circulating tumor cells (CTCs) are cancer cells that are shed from the primary and metastatic tumor(s). They can be detected in peripheral blood samples using different technologies, but their identification and characterization require extremely sensitive and specific analytical methods [2,3,4,5,6]. Their analysis is considered as a real-time liquid biopsy for patients with cancer [7,8,9,10]. In 2011, the U.S. Food and Drug Administration (FDA) cleared the CellSearch^®^ system (Menarini Silicon Biosystems) for CTC analysis to monitor patients with metastatic breast, colorectal and prostate cancer [11,12,13]. The CellSearch^®^ epithelial cell-based assay has clearly demonstrated its clinical significance and is now used as the gold standard in clinical studies evaluating different cancer types. Even though a very limited number of studies have evaluated melanoma CTCs using the CellSearch^®^ Circulating Melanoma Cell Kit, they all provided similar results, reflecting the robustness and reproducibility of this assay. The detection of circulating melanoma cells (CMCs) was described for the first time in 1991. Since then, the many studies on CMCs from patients with melanoma at different stages and using different detection approaches have reported conflicting results [14]. Indeed, metastatic melanoma is a highly heterogeneous tumor and CMCs may display different phenotypes and functional states.

Moreover, CMC analysis with the CellSearch^®^ detection kit does not allow discriminating between dead and viable CMCs, the only CMCs involved in metastatic development [15]. The functional EPithelial ImmunoSPOT (EPISPOT) assay was described in 2005 and allows the identification of viable CTCs in peripheral blood samples of patients with cancer (e.g., breast, prostate, and colon cancer) [16,17,18,19,20] by detecting proteins secreted/released/shed by single viable epithelial cancer cells [21].

The aim of this study was to compare CMC detection using the CellSearch^®^ system and a new EPISPOT assay (S100-EPISPOT assay) designed to identify viable CMCs that secrete S100, a protein expressed and secreted by melanoma cells [22], in blood samples from patients with metastatic melanoma.

## 2. Materials and Methods

### 2.1. Patient Cohort

A prospective controlled observational comparative study (Circulating Tumor Cells and Melanoma: Comparing the EPISPOT and CellSearch Techniques; NCT01558349) was conducted at the Nîmes University Hospital, Nîmes, France, between June 2013 and June 2017. The main objective was to determine if we can observe more positive patients with the EPISPOT assay than the CellSearch^®^ system. All patients with melanoma signed a written informed consent before enrolment in the CELLCIRC study and treatment initiation. The study was carried out in accordance with the World Medical Association Declaration of Helsinki. The experimental protocol was approved by the French bioethical committee “Sud Méditerranée III” (Approval reference No. 2012.06.10). Blood samples from healthy volunteers (*n* = 38) and patients with metastatic malignant melanoma (*n* = 50; before any treatment) were collected in the morning and processed within 24 h.

### 2.2. Melanoma Cell Lines

The melanoma cancer cell lines WM-266-4 (ATCC^®^ CRL-1676™) and MV3 (kindly provided by Klaus Pantel, University of Tumor Biology, Hamburg, Germany) were used for optimizing the S100-EPISPOT assay. WM-266-4 cells were maintained in αMEM medium (22571, Gibco, Grand Island, USA) supplemented with 10% fetal calf serum (FCS), and MV3 cells in RPMI 1640 medium (L0501, Dominique Dutscher, Brumath, France), supplemented with 5mM L-glutamine (25030, Gibco, Grand Island, USA) and 10% FCS.

### 2.3. Flow Cytometry Experiments

Intracellular expression of the S100 protein in WM-266-4 and MV3 cells was determined by flow cytometry using a Cyan cytometer (Beckman-Coulter, Villepinte, France) and a fixation/permeabilization kit (Beckman Coulter, Brea, USA). The two anti-S100 antibodies (clones 8B10 and 6G1) used in the EPISPOT assay were tested to confirm S100 expression in these melanoma cell lines.

### 2.4. Immunofluorescence Assay

Melanoma cell lines were immunostained with the two anti-S100 antibodies (8B10 and 6G1), as described for the flow cytometry experiments. Then, cells were seeded on glass slides using a Cytospin 4 centrifuge (Shandon, Runcorn, England) and mounted with ProLong Gold Antifade reagent with 4’,6-diamidino-2-phenylindole (DAPI) (Invitrogen). S100 expression was analyzed with a fluorescent microscope (Axio Imager M1, Carl Zeiss Vision, Halbermoos, Germany).

### 2.5. S100-EPISPOT Assay

As CMCs are rare in peripheral blood, a pre-enrichment step was performed before the EPISPOT assay. To separate erythrocytes and leukocytes from CMCs, the RosettSep^TM^ reagent (20 μL/mL) was added to 13–15 mL of blood collected in EDTA tubes, and enrichment was performed following the manufacturer’s instructions (RosetteSep^TM^ CTC Enrichment Cocktail containing Anti-CD36, STEMCELL Technologies). During the S100-EPISPOT assay, enriched CMCs were cultured on a membrane coated with the anti-S100 8B10 antibody (10 ng/μL; Abcam) for 2 days. Then, secreted S100 captured by the 8B10 antibody was detected by incubation with another antibody against S100 (6G1: 3 ng/µL; Abcam) conjugated to AlexaFluor 488. Single fluorescent S100 immunospots were counted under a fluorescent microscope equipped with a camera and computer-assisted analysis (KS ELISPOT, Carl Zeiss Vision). The detailed procedure of the EPISPOT assay has been described by Soler et al. [21]. Results were corrected as “number of cells per 7.5 mL of blood” to be comparable with those obtained with the CellSearch system.

### 2.6. Cell Search^®^ System

Patient blood samples were collected in special CellSave^®^ tubes and the analysis was performed using the Circulating Melanoma Cell Kit (9594V, Menarini, Bologna, Italy), according to the manufacturer’s instructions, as previously described [23]. Reagents consisted of ferrofluids coated with anti-CD146 antibodies to enrich melanoma cells and endothelial cells, a phycoerythrin-conjugated antibody that binds to high molecular weight melanoma-associated antigen (HMW-MAA) to identify melanoma cells, a mixture of two allophycocyanin-conjugated antibodies against CD45 to identify leukocytes and against CD34 to identify endothelial cells, and the nuclear dye DAPI to identify nucleated cells. The criteria to identify an object as a melanoma cell included: round to oval morphology, visible (DAPI-positive) nucleus, positive staining for HMW-MAA, and negative staining for CD45 and CD34. Results were expressed as number of cells per 7.5 mL of blood.

### 2.7. Statistical Analysis

The sensitivity and specificity of each technology (S100-EPISPOT and CellSearch^®^) were assessed by comparing the true positive/negative and false positive/negative results for healthy donors and patients with melanoma using the McNemar’s test and the McNemar’s test with continuity correction.

Patients’ characteristics were described using medians and ranges (quantitative variables). The Fisher’s test was used to evaluate the association between categorical clinical characteristics and CMC detection. The association between lactate dehydrogenase (LDH) value and CMC number was assessed using Spearman and Kendall rank correlation coefficients.

The median OS was analyzed using the Kaplan–Meier method. The end point was death by any cause. Survival curves were compared with the non-parametric log rank test (*p* ≤ 0.05 considered as significant). The univariate Cox proportional hazard regression model was used to estimate the hazard ratio and 95% confidence intervals (CI) for the CellSearch^®^ assay, and the risk of death in function of the CMC number (<2 vs. ≥2) after verifying the hazard proportional test.

Statistical analyses were performed using SAS, version 9.4 (SAS Institute).

## 3. Results

### 3.1. S100 Expression in Melanoma Cell Lines

**Figure 1:**
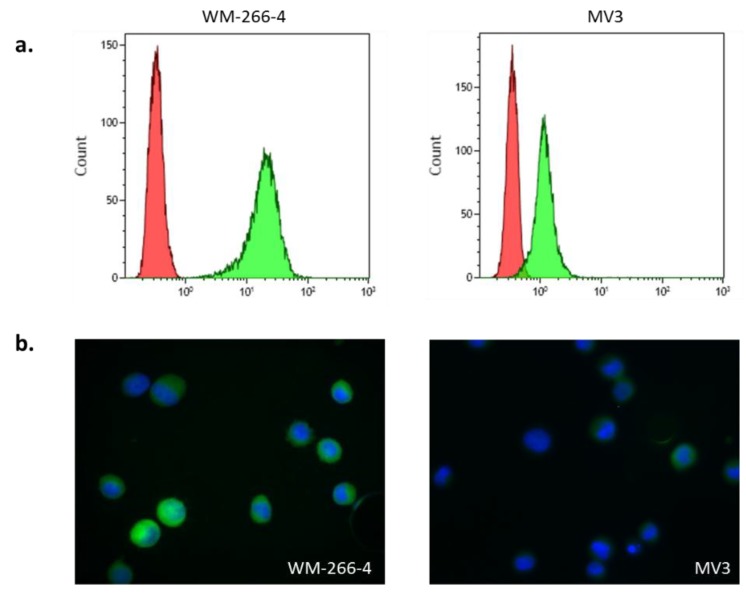
S100 protein expression in WM-266-4 and MV3 cells by (**a**) flow cytometry and (**b**) immunofluorescence analysis using the anti-S100 antibody 6G1 conjugated to AlexaFluor 488.

To develop a new EPISPOT assay for CMC detection, two different melanoma cell lines (WM-266-4 and MV3), derived from metastatic sites, were used. Analysis of S100 protein expression by flow cytometry (FC) showed that both cell lines expressed S100, but at different levels (one log difference between cell lines) (Figure 1a). These results were confirmed by immunofluorescence (IF) experiments (Figure 1b). Both cell lines expressed S100, but the signal intensity was higher in WM-266-4 than in MV3 cells (same exposure time). Similar results were obtained with the two anti-S100 antibodies (8B10 and 6G1) used in the S100-EPISPOT assay. These findings indicated that both melanoma cell lines could be used to optimize the S100-EPISPOT assay.

### 3.2. S100-EPISPOT Assay

**Figure 2:**
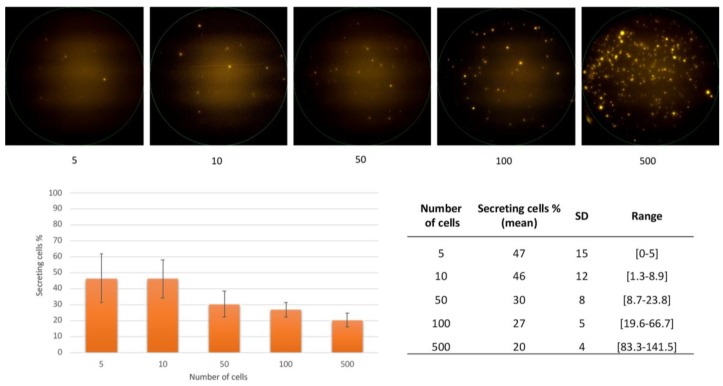
Melanoma cell detection using the S100-EPISPOT assay. Representative images of immunospots using WM-266-4 cells (upper panels) and percentage (*n* = 3) of S100-secreting cells recovered from serial dilutions of WM-266-4 cells (lower panels). SD = standard deviation.

Then, the two melanoma cell lines were used to evaluate the feasibility and detection threshold of the S100-EPISPOT assay. No immunospots (indicative of S100 secretion) could be detected when using MV3 cells, possibly due to their very weak S100 expression, as observed by FC and IF. Conversely, on average, 20–47% of WM-266-4 cells secreted S100 (Figure 2). This detection rate is considered normal for cell lines analyzed with the EPISPOT assay because of variations in cell cycle and protein productivity. In parallel, enriched peripheral blood mononuclear cells (PBMCs) from healthy donors were tested (*n* = 3) to confirm that S100 was not secreted by PBMCs (data not show) and validate this assay for CMC detection after blood enrichment.

Surprisingly, the detection rate was higher with smaller numbers of tested cells (Figure 2). These data demonstrate that the new S100-EPISPOT assay could be used to detect rare CMCs in peripheral blood. Previous studies also reported a better recovery rate for small number of cancer cells spiked in samples of healthy donors (1–20 cancer cells/10 mL) [24,25].

### 3.3. Specificity of the S100-EPISPOT Assay

**Figure 3:**
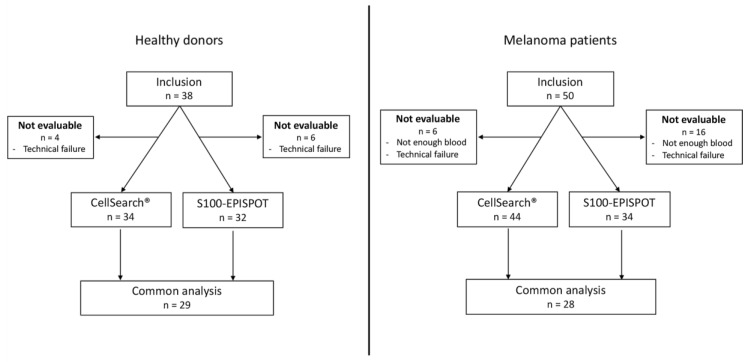
Study flowchart showing the number of included healthy donors and patients and the number of patients and healthy donors who underwent circulating melanoma cell (CMC) analysis using the S100- EPISPOT assay and CellSearch^®^ system.

Among the 38 healthy donors enrolled, six and four donors could not be evaluated by EPISPOT and CellSearch, respectively, for reasons listed in Figure 3a. The specificity of the S100-EPISPOT and CellSearch^®^ assays were compared using blood samples from 38 healthy donors and two detection thresholds (≥1 and ≥2 CMCs) (Table 1a). The CellSearch^®^ system gave similar results with both thresholds: 94% and 97%. The S100-EPISPOT assay specificity was lower for the ≥1 CMC threshold (78%), but reached 97%, as for CellSearch^®^, for the ≥2 CMC threshold. These results are consistent with previous reports and allowed for choosing a cut-off of 2 CMCs for the positive CMC detection [23,26].

### 3.4. Circulating Melanoma Cell Detection in Patients with Metastatic Melanoma

Among the 50 patients enrolled, 16 and 6 could not be evaluated by EPISPOT and CellSearch, respectively, for reasons listed in Figure 3b. The patients included in this study (26 men and 24 women with a mean age of 64, range, 29–89) had metastatic melanoma, with a median survival of 6.72 months (95% CI = 4.24–12.16). CMCs were detected using the CellSearch^®^ system and the S100-EPISPOT assay in blood samples from 44 and 34 patients, respectively. Using the cut-off of ≥2 CMCs per 7.5 mL of blood, 10/44 (23%) patients were positive for CMCs with the CellSearch^®^ system, and 15/34 (44%) with the S100-EPISPOT assay (Table 2). The S100-EPISPOT assay gave a higher number of positive patients than the CellSearch^®^ system, but with a smaller range: EPISPOT (0–450) and CellSearch^®^ (0–4937). Analysis of the specificity and sensitivity using the results obtained for samples from healthy donors (*n* = 28) and patients with metastatic melanoma (*n* = 29) tested with both assays, showed that sensitivity was 48% for S100-EPISPOT and 21% for CellSearch^®^ (*p* = 0.0114, corrected *p* = 0.0269), and specificity was 100% for S100-EPISPOT and 96% for CellSearch^®^ (Table 1b). The correlation between both technologies was assessed using the Spearman (*r_s_* = 0.49, *p* = 0.0070) and Kendall (τ = 0.44, *p* = 0.0055) tests.

### 3.5. Clinical Relevance of CMC Detection

No significant association between the patients’ clinical characteristics and CMC detection with EPISPOT and CellSearch^®^ was observed (Table 3), except for high LDH level (2-fold higher than normal) and CMC detection using CellSearch^®^ (*p* = 0.0315). The correlation between LDH level and CTC detection by CellSearch^®^ was significant using the Spearman (*r_s_* = 0.39, *p* = 0.0138) and Kendall (τ = 0.30, *p* = 0.0132) tests.

**Figure 4:**
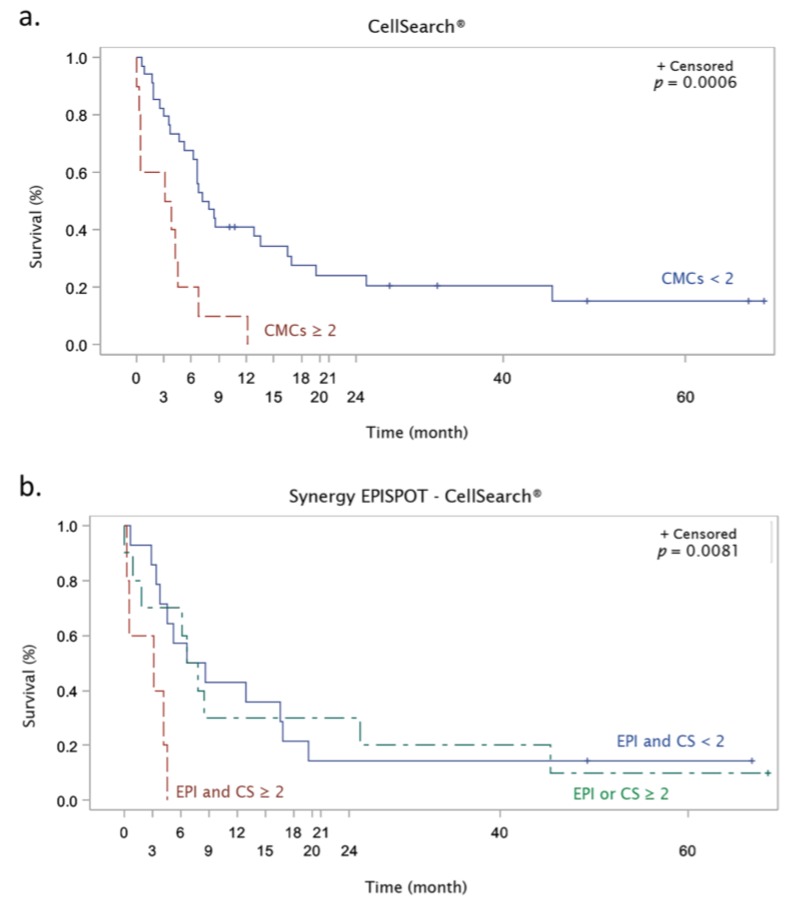
Kaplan–Meier survival curves of patients with metastatic melanoma according to the detection cut-off of 2 CMCs using the (**a**) CellSearch^®^ system, and (**b**) when combining the S100-EPISPOT (EPI) and CellSearch^®^ (CS) assay results.

In univariate Kaplan–Meier analyses, OS was associated only with CMC detection by CellSearch^®^ (CS) (*p* = 0.0006) (Figure 4a). Hazard ratio for death was 3.57 (95% CI 1.64–7.77; Cox regression analysis with CMC <2 vs. ≥2). No association was detected between OS and the classical clinical variables (age, sex, Breslow depth, Clark stage, LDH, BRAF mutation, and number of metastatic sites) (Table S1). Moreover, no significant association was observed for the S100-EPISPOT assay (EPI) alone (Figure S1). Analysis of the potential synergistic effect of combining both assays indicated that the “double negative” (EPI and CS < 2 CMCs) group was not associated with OS (*p* = 0.3419) compared with the “double and/or simple positive” (EPI and/or CS ≥2) group. Conversely, analysis of the “double positive” (EPI and CS ≥ 2 CMCs)*,* “simple positive” (EPI or CS ≥2 CMCs) and “double negative” (EPI and CS < 2 CMCs) groups highlighted the association of the “double positive” group with OS (*p* = 0.0081) (Figure 4b).

## 4. Discussion

The cancer metastatic cascade is a complex process characterized by several events, including cell migration, local invasion, intravasation of tumor cells into the circulation and extravasation at distant sites to form detectable metastases [27]. The mechanisms involved in this multistep process are largely unknown, but recent studies suggested that some CTCs could be at the origin of distant metastases [28,29]. For this reason, CTC detection has become a great challenge for the personalized treatment of patients with cancer.

In the literature, the number of techniques used for CTC detection in patients with melanoma is almost equal to the number of studies published on this topic. These methods are based on the physical or phenotypical properties of melanoma cells [3,30,31]. Up to now, none of them has been validated because of the lack of reproducible results. Nestin, CD133 [32], receptor activator of NF-k B (RANK) [33], ATP-binding cassette sub-family B member 5 (ABCB5) [34], CD20 [35], and CD271 [36] have been identified as potential candidates for the identification of melanoma-initiating cells.

Compared with CTC detection techniques based only on the expression of surface markers, assays that identify viable/functional cells could be more interesting and more relevant for monitoring the response to treatment. Functional CTC analysis also offers the possibility to determine the biological properties of metastatic cells, including the identification of metastasis-initiating cells [37]. Currently, the EPISPOT assay is the only functional assay to detect viable, prognostically relevant CTCs at the single-cell level after enrichment by leukocyte depletion. This system has been used to test blood samples from hundreds of patients with different tumor types [17,18,19]. As this technique could be considered to be a protein secretion-profiling assay, the analyzed proteins should be specific and significantly produced and released by tumor cells. Among the proteins expressed and released by melanoma cells, the S100 family is the most studied [22,38]. S100B expression is increased in melanoma cells compared with melanocytes and can be used for the diagnosis of metastatic malignant melanoma by immunohistochemistry. Moreover, serum S100B level increases in patients with melanoma, independently of the cancer stage. Its expression is clearly correlated with the presence of metastases, tumor burden, prognosis, and survival [39,40]. Recent data demonstrated that isolated CMCs obtained from patients with metastatic melanoma uniformly express S100 [41].

In this study, we first described the new S100-EPISPOT assay to detect viable CMCs, and then compared its results with those obtained with the CellSearch^®^ system (Circulating Melanoma Cell Kit). In two melanoma cell lines, we found that S100 expression and secretion were heterogeneous. Consequently, only the cell line that strongly expresses S100 (WM-266-4 cells) secreted enough protein to be detected by the S100-EPIPSOT assay at the single-cell level. The subpopulation of cancer cells that weakly express S100 are missed using this assay. Moreover, WM-266-4 cell detection by the S100-EPISPOT system was more efficient when only a few cells were present in the sample. These experimental observations were confirmed by data obtained from blood samples from a homogeneous group of patients with metastatic melanoma, indicating that the S100-EPISPOT assay sensitivity is significantly higher than that of the CellSearch® system. Specifically, the percentage of patients with ≥2 CMCs was 48% with the S100-EPISPOT assay and 21% with the CellSearch^®^ system (corrected *p* = 0.0269).

CMC detection (both methods) was not associated with Breslow depth and BRAF mutation status. Only LDH value was significantly correlated with CMC detection (CellSearch^®^ method) in Spearman (*r_s_* = 0.39, *p* = 0.0138) and Kendall (*k* = 0.30, *p* = 0.0132) tests.

Analysis of the prognostic value of CMC detection (both assays) indicated that only CMC ≥ 2 with CellSearch^®^ was significantly associated with the OS (*p* = 0.006), as previously reported for the CellSearch^®^ CMC detection system. By using this CellSearch^®^ system, Rao et al. [23] detected at least two CMCs in 23% (18/79) of blood samples from 44 patients with metastatic melanoma. They also found that OS was shorter in patients with ≥2 CMCs per 7.5 mL of whole blood compared with the <2 CMC group. Similarly, Khoja et al. [26] showed that 26% of patients with metastatic skin melanoma had more than two CMCs per 7.5 mL of blood, and that the median OS was significantly shorter in this group.

In our patient cohort, no other clinical features, such as number of metastatic sites, Breslow depth, BRAF mutational status and even LDH level (routinely measured in the clinical practice) provided prognostic information. The S100-EPISPOT assay results alone did not predict the patients’ clinical outcome, although this test was more sensitive than the CellSearch^®^ system. Conversely, by combining the data obtained by both methods, OS was significantly associated (*p* = 0.0005) with “double positive results” (CellSearch^®^ and S100-EPISPOT) compared with “simple positive result” (CellSearch^®^ or EPISPOT) and “double negative result” (CellSearch^®^ and S100-EPISPOT). This suggests that OS is shorter in patients in whom CMCs are detected with both methods.

However, in our patients with advanced disease and short survival probability (0.38, 95% CI 0.25–0.51 after 12 months and 0.16, 95% CI 0.07–0.27 after 24 months), measuring OS is not of clinical interest. The crucial aim of liquid biopsy is to obtain reliable and “real-time” information before, during, and after treatment to monitor the patient response. This is especially important for melanoma because currently no melanoma-specific blood-based biomarker is available for routine use in clinical practice. Another circulating biomarker of interest in advanced melanoma is ctDNA. The majority of studies have been applied to patients treated with BRAF inhibitors, via monitoring of the singular BRAFV600 mutation, predicting response to therapy and prognosis in metastatic melanoma [42]. Nevertheless, clinical trials that look at patient outcome as a result of ctDNA-guided clinical decisions are required before ctDNA can be successfully established as a melanoma-specific biomarker in clinical practice.

Finally, the main interest of our study is the finding that the new S100-EPISPOT assay has a good sensitivity (48%) for CMC detection and an acceptable specificity. It would be interesting to determine whether the S100-EPISPOT functional assay could be used for the early detection of tumor relapse or for monitoring therapy response in patients with non-metastatic melanoma.

Moreover, a completely new optimized EPISPOT assay, named EPIDROP (EPISPOT in a DROP) [43], is currently under development. This innovative micro-droplet technology allows not only the detection of viable S100-secreting CMCs at the single-cell level, but also the immunostaining of all CMCs before their encapsulation, for instance for the identification of tumor cells that could be targeted by immunotherapy (e.g., PD-L1-positive). This technology represents a new combination of standard CMC detection by IF, like the CellSearch^®^ system, with a functional assay to identify the subset of functional and potentially metastasis-competent CMCs.

## Figures and Tables

**Table 1:** (**a**) Specificity of the S100-EPISPOT and CellSearch CMC assays in healthy donors using two different CMC cut-offs. (**b**) Sensitivity and specificity of the two assays with the cut-off ≥2 CMCs (healthy donors: *n* = 28; patients with metastatic melanoma: *n* = 29).

a.						
	**S100-EPISPOT (n = 32)**			**CellSearch CMC (n = 34)**		
**<1**	25		(78%)	32	(94%)	
**≥1**	7		(22%)	2	(6%)	
**<2**	31		(97%)	33	(97%)	
**≥2**	1		(3%)	1		
**b.**						
		**S100 EPISPOT**		**CellSearch CMC**	*p value* (Mac Nemar)	*p value* (corrected Mac Nemar)
**Sensitivity** (n = 29)		48%		21%	**0.0114**	**0.0269**
**Specificity** (n = 28)		100%		96%	0.3173	1

**Table 2:** Description of CMC detection for the S100-EPISPOT and CellSearch^®^ assays in (**a**) all patients included in the study and (**b**) the subpopulation of patients tested with both assays.

a.										
**Nb Patients**	**Assay**	**Total**	**Failed**	**Mean**	**Standard Deviation**	**Median**	**Min**	**Max**	**Lower quartile**	**Upper quartile**
50	S100-EPISPOT	34	16	21.59	80.35	1.00	0.00	450.00	0.00	3.00
	CellSearch®	44	6	142.57	752.01	0.00	0.00	4937.00	0.00	1.00
**b.**										
**Nb Patients**	**Assay**	**Total**	**Failed**	**Mean**	**Standard Deviation**	**Median**	**Min**	**Max**	**Lower quartile**	**Upper quartile**
29	S100-EPISPOT	29	0	25.24	86.68	1.00	0.00	450.00	0.00	4.00
	CellSearch®	29	0	206.10	924.77	0.00	0.00	4937.00	0.00	1.00

**Table 3:** Clinical characteristic of patients with metastatic melanoma in function of CTC detection with the S100-EPISPOT and CellSearch^®^ assays.

	EPISPOT			CellSearch®		
	**< 2**	**≥ 2**	***p* value (Fisher)**	**< 2**	**≥ 2**	***p* value (Fisher)**
	**n = 19**	**n = 15**		**n = 34**	**n = 10**	
**Sex**						
Men	8	9	0.4905	18	4	0.7205
	(42.11%)	(60.00%)		(52.94%)	(40.00%)	
Women	11	6		16	6	
	(57.89%)	(40.00%)		(47.06%)	(60.00%)	
**BRAF mutation**						
No	8	6	1.0000	15	2	0.4267
	(47.06%)	(54.55%)		(48.39%)	(28.57%)	
Yes	9	5		16	5	
	(52.94%)	(45.45%)		(51.61%)	(71.43%)	
**Ulceration**						
Absence	4	6	0.0656	11	4	0.6618
	(36.36%)	(85.71%)		(52.38%)	(66.67%)	
Presence	7	1		10	2	
	(63.64%)	(14.29%)		(47.62%)	(33.33%)	
**Metastatic sites**						
Nb ≤ 2	10	7	1.0000	22	3	0.0738
	(52.63%)	(46.67%)		(64.71%)	(30.00%)	
Nb > 2	9	8		12	7	
	(47.37%)	(53.33%)		(35.29%)	(70.00%)	
**LDH value**						
Normal	6	5	0.2909	10	1	**0.0315**
	(42.86%)	(35.71%)		(34.48%)	(10.00%)	
> Normal	7	4		16	4	
	(50.00%)	(28.57%)		(55.17%)	(40.00%)	
2x > Normal	1	5		3	5	
	(7.14%)	(35.71%)		(10.34%)	(50.00%)	
**Primary tumor localization**						
Other	1	6	**0.0106**	6	3	0.2017
	(2.94%)	(17.65%)		(13.95%)	(6.98%)	
Face	1	0		0	1	
	(2.94%)	(0.00%)		(0.00%)	(2.33%)	
Lower limb	8	2		12	1	
	(23.53%)	(5.88%)		(27.91%)	(2.33%)	
Upper limb	6	1		7	1	
	(17.65%)	(2.94%)		(16.28%)	(2.33%)	
Torso	3	6		9	3	
	(8.82%)	(17.65%)		(20.93%)	(6.98%)	

## Supplementary Materials

The following are available online at https://www.mdpi.com/2073-4409/8/7/755/s1. Table S1: Univariate analysis of overall survival in patients with metastatic melanoma using the Kaplan–Meier method, Figure S1: Kaplan–Meier survival curves of patients with metastatic melanoma according to the detection cut-off of two CMCs using the S100-EPISPOT (EPI).
