# Supplementary material for: S100-EPISPOT: A New Tool to Detect Viable Circulating Melanoma Cells
Source: Cells. 2019 Jul 20;8(7):755. doi: 10.3390/cells8070755 (PMC6678250; doi:10.3390/cells8070755)
Supplement: Supplementary file 1 [file cells-08-00755-s001.pdf]

Supplementary Materials: The following are available online at www.mdpi.com/xxx/s1.

Table S1. Univariate analysis of overall survival in patients with metastatic melanoma using the Kaplan–Meier method.

	n	Median estimate	IC 95 %		*p* value (Log Rank)
**Age, years (median)**	**50**	.	.	.	0.2936
< 64.50	25	6.735	3.483	8.674	
≥ 64.50	25	6.669	3.745	14.324	
**Sex**	**50**	.	.	.	0.9791
Women	26	6.719	3.483	13.142	
Men	24	6.702	2.957	14.324	
**Breslow depth (median)**	**41**	.	.	.	0.0578
< 2.90	19	8.674	2.957	25.166	
≥ 2.90	22	4.928	2.497	6.735	
**Clark stage**	**30**	.	.	.	0.0822
III	5	19.680	6.209	.	
IV	20	4.419	2.497	8.674	
V	5	6.735	0.493	45.372	
**LDH value**	**42**	.	.	.	0.2699
2x > Normal	8	2.743	0.066	8.509	
> Normal	22	6.998	3.483	14.324	
Normal	12	6.423	1.873	25.166	
**BRAF mutation**	**42**	.	.	.	0.4112
No	20	4.058	1.676	13.503	
Yes	22	7.573	5.224	13.142	
**Ulceration**	**28**	.	.	.	0.8696
Absence	15	4.632	0.887	8.509	
Presence	13	6.735	3.745	13.503	
**Nb metastatic sites**	**49**	.	.	.	0.8749
1	10	3.795	0.263	.	
2	15	7.261	2.497	16.559	
3	11	6.735	2.661	16.887	
4	8	5.224	0.394	19.680	
5	5	6.735	0.493	13.142	
**EPISPOT (EPI)**	**34**	.	.	.	0.7416
< 2 CMCs	19	6.637	2.957	16.559	
≥ 2 CMCs	15	6.209	0.887	8.509	
**CellSearch® (CS)**	**37**	.	.	.	**0.0006**
< 2 CMCs	27	7.573	5.224	16.559	
≥ 2 CMCs	10	3.499	0.066	4.600	
**SYNERGIE EPI/CS**	**29**	.	.	.	0.3419
EPI and CS < 2	14	7.655	3.483	16.887	
EPI and/or CS ≥ 2	15	4.600	0.493	7.885	
**SYNERGIE EPISPOT/CS**	**29**	.	.	.	**0.0081**
EPI and CS < 2	14	7.655	3.483	16.887	
EPI and CS ≥ 2	5	3.154	0.263	4.600	
EPI or CS ≥ 2	10	7.277	0.066	25.166	

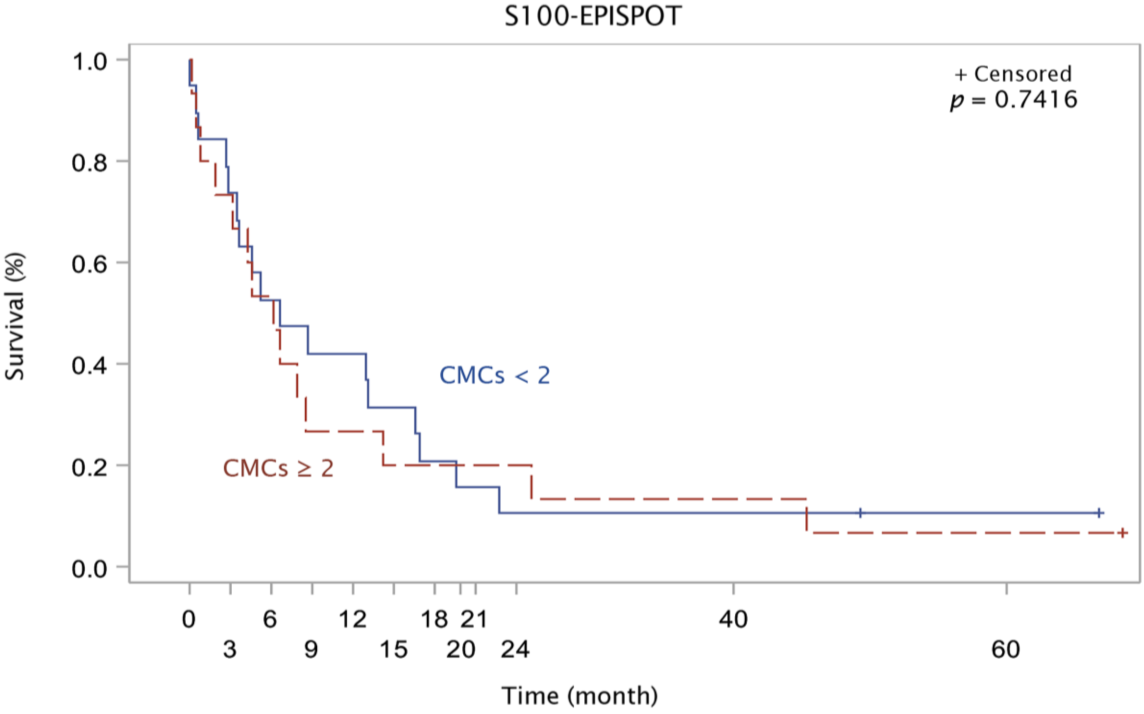

Figure 1. Kaplan–Meier survival curves of patients with metastatic melanoma according to the detection cut-off of two CMCs using the S100-EPISPOT (EPI).
